# Lanadelumab safety, efficacy, and pharmacokinetics in patients aged ≥12 with hereditary angioedema in China: an open-label, multicenter study

**DOI:** 10.3389/fimmu.2026.1750735

**Published:** 2026-04-29

**Authors:** Yuxiang Zhi, Rongfei Zhu, Yuemei Sun, He Lai, Hong Ren, Yi Wang, Lin Dong

**Affiliations:** 1 Department of Allergy, Peking Union Medical College Hospital, Chinese Academy of Medical Sciences, Beijing, China; 2 Department of Allergy, Tongji Hospital, Tongji Medical College, Huazhong University of Science & Technology, Wuhan, China; 3 Department of Allergy, Yantai Yuhuangding Hospital, Yantai, Shandong, China; 4 Department of Allergy, The Second Affiliated Hospital of Guangzhou Medical University, Guangzhou, Guangdong, China; 5 Takeda Development Center Americas, Inc., Cambridge, MA, United States; 6 Takeda APAC Biopharmaceutical Research and Development Company Limited, Shanghai, China

**Keywords:** Chinese patients, HAE long-term prophylaxis, hereditary angioedema, lanadelumab, pharmacodynamics, pharmacokinetics, phase 3, plasma kallikrein inhibitor

## Abstract

**Background:**

Lanadelumab is a plasma kallikrein inhibitor approved in many countries, including China, for the long-term prophylaxis of hereditary angioedema (HAE). The primary objective was safety evaluation of lanadelumab for HAE long-term prophylaxis in a Chinese patient population. Secondary objectives included efficacy, pharmacokinetics, pharmacodynamics, and immunogenicity evaluations.

**Methods:**

This open-label, prospective, phase 3, post-approval commitment study included patients in China aged ≥12 years with HAE due to C1 inhibitor deficiency (HAE-C1INH) (NCT05460325). Eligible patients with ≥1 HAE attack in the 4-week run-in period received subcutaneous lanadelumab 300 mg every 2 weeks during days 0–182 for 14 doses in total.

**Results:**

Twenty patients were enrolled (median age, 38 years; 7 male), and all completed the study. No treatment-emergent adverse events (TEAEs) were fatal, led to treatment discontinuation, or were of special interest. Fifteen (75%) patients reported ≥1 TEAE during the study, most commonly COVID-19 infection (50%). Nine (45%) patients reported treatment-related TEAEs, most frequently injection site pain (20%) and swelling (15%). Injection site reactions occurred in 25% of patients. Mean ± SD monthly rate of investigator-confirmed attacks decreased by 99.1% during days 0–182 versus run-in (0.04 ± 0.14 vs 2.50 ± 1.44). Eighteen (90%) patients were attack free during days 0–182; 2 patients experienced a total of 4 mild and 1 moderate investigator-confirmed HAE attacks. Lanadelumab pharmacokinetics, pharmacodynamics, and immunogenicity in Chinese patients were consistent with other patient populations.

**Conclusion:**

These findings in patients with HAE in China are consistent with the profile of lanadelumab established in patients from other regions, supporting its use as first-line long-term prophylaxis.

## 1 Introduction

Hereditary angioedema (HAE) is a rare genetic disorder that manifests in unpredictable, painful, and potentially debilitating cutaneous and/or subcutaneous edema commonly affecting the face, abdomen, limbs, and/or genitalia; attacks with laryngeal involvement can be fatal due to the risk of asphyxiation (1, 2). Most cases of HAE are caused by mutations in *SERPING 1*, resulting in diminished levels of circulating C1 inhibitor (C1INH; HAE-C1INH-Type1) or dysfunctional C1INH (HAE-C1INH-Type2) (1, 3). Attenuated C1INH activity leads to bradykinin overproduction and subsequent uncontrolled bradykinin B2 receptor activation, promoting extravasation and eventually precipitating sudden swelling attacks seen with HAE.

The estimated global prevalence of HAE is approximately 1:50,000, although the true prevalence remains uncertain, as the lack of awareness and limited access to diagnostics often lead to delayed diagnosis (2, 4). A 2024 systematic review of 65 publications estimated the overall prevalence of HAE to range from 0.13 to 1.6 cases per 100,000, with mean or median delay in diagnosis ranging from 3.9 to 26 years (5). In China, the true prevalence of HAE is unknown (6) and regional variations have been reported, likely attributed to differences in medical practices and access to diagnostics (7). A 2022 international survey found that <1,000 patients have been diagnosed (2, 4), whereas approximately 28,000 patients with HAE are potentially undiagnosed (6, 8). Other studies estimated a mean diagnosis delay of 11.0 to 12.6 years in China (8, 9); however, there is a trend of decreasing HAE diagnostic delay in China (7).

Optimal HAE management, as outlined by the recently updated 2024 Chinese expert consensus and in line with other international guidelines, includes on-demand treatment for acute attacks, short-term prophylaxis therapy before exposure to HAE attack−inducing events (eg, medical or dental procedures), and long-term prophylaxis (LTP) therapy to reduce attack frequency. The goal of treatment is to achieve complete disease control and normalization of patients’ lives (1, 10–12). A notable update included in the Chinese expert consensus was the recommendation of several first-line LTP therapies including lanadelumab, the only first-line LTP therapy approved in China to date (7, 10, 12). Until recently, LTP therapies available to Chinese patients were limited to attenuated androgen derivatives and antifibrinolytics (7, 13), both of which have since been relegated to second-line due to their side-effect profile and uncertain efficacy, respectively (1, 10).

Lanadelumab is a human monoclonal antibody inhibitor of plasma kallikrein approved for LTP in patients with HAE in >60 countries, including the United States and Europe (14, 15). Regulatory approval was granted in China in December 2020 (2, 16). The safety and efficacy of lanadelumab were established in several clinical trials (17–20), including the phase 3, placebo-controlled HELP Study (NCT02586805) and the open-label extension study (HELP OLE; NCT02741596), which cumulatively enrolled more than 200 patients across both studies (17, 19). Three large, phase 4, non-interventional studies, the EMPOWER, ENABLE, and INTEGRATED real-world studies, which cumulatively enrolled more than 400 patients, further demonstrated the long-term safety and effectiveness of lanadelumab in clinical practice (21–23). In addition, the pharmacokinetics and pharmacodynamics have also been characterized (18, 24, 25). However, the aforementioned data were collected from patients outside of China, predominantly in the United States, Europe, and Japan (17–20, 26). Published data on the outcomes of Chinese patients receiving lanadelumab are limited to a case study (27) and 3 small single-center studies (28–30), and more recently, a real-world study across 5 centers (31). The observation that clinical features of HAE, such as primary attack locations, may differ between White and Chinese populations (13) highlights the importance of collecting data specific to Chinese patients. To support lanadelumab dosing in Chinese patients, a prospective, phase 3, post-approval commitment study was conducted to evaluate the safety, efficacy, pharmacokinetics, pharmacodynamics, and immunogenicity of lanadelumab treatment for 6 months in Chinese patients with HAE due to C1INH deficiency (HAE-C1INH).

## 2 Methods

### 2.1 Study design and treatment

This was an open-label, prospective, single-arm, multicenter study in patients with HAE-C1INH from China (NCT05460325; first registered July 13, 2022), conducted in compliance with institutional review board/independent ethics committee regulations per Good Clinical Practice guidelines, in accordance with the principles of the Declaration of Helsinki and all applicable local regulations. This study was approved by the Drug Clinical Trial Ethics Committee of Peking Union Medical College Hospital, Chinese Academy of Medical Sciences (No. KS2022050), Clinical Trial Ethics Committee of the Second Affiliated Hospital of Guangzhou Medical University (No. Y2022-15-01), Clinical Trial Ethics Committee of Huazhong University of Science and Technology ([2022] No. 28), and the Yantai Yuhuangding Hospital Drug Clinical Trial Ethics Committee ([2022] No. 18-01). Informed consent/assent (as applicable) was obtained for all enrolled patients.

The study included a screening period (≤4 weeks), a 2-week washout period, a 4- to 8-week run-in period before lanadelumab initiation, and a 26-week lanadelumab treatment period, during which patients received lanadelumab 300 mg subcutaneously every 2 weeks (q2w). Patients who completed the treatment period were to receive 14 doses of lanadelumab in total during days 0–182 (± 3 days). Patients were followed for an additional 4 weeks upon completion of the treatment period.

Per the protocol, adult patients aged ≥18 years who were receiving any LTP for HAE (eg, androgens, anti-fibrinolytics, or C1INH replacement therapy [the latter of which was not available in China at the time of the study]) were required to undergo a 2-week washout period, during which icatibant was used as an on-demand therapy. Patients underwent the LTP washout only when deemed safe to do so by the study investigators. LTP washout was not required in adolescent patients (aged 12 to <18 years). Patients who failed screening due to a single laboratory test result that did not meet eligibility criteria could have had that laboratory test repeated at the discretion of the investigator. This included a repeat of only the failed assessment rather than a repeat of all screening assessments. Screened patients who were not receiving LTP therapy for HAE or who had completed the required washout period were then enrolled and entered a run-in period of 4 to 8 weeks to determine their baseline attack rate. In patients who did not experience ≥1 investigator-confirmed attack after 4 weeks, the run-in period was extended by 4 weeks, during which time they had to have experienced ≥2 investigator-confirmed attacks in 8 weeks to be eligible for study participation. Patients who experienced ≥3 investigator-confirmed attacks before the end of the 4 weeks exited the run-in period early and proceeded to the treatment period.

Therapies for co-existing conditions, including short-term prophylaxis, if medically indicated, and treatments for any adverse events (AEs) were permitted; however, the following treatments were not permitted during the study: LTP for HAE, angiotensin-converting enzyme (ACE) inhibitors, estrogen-containing medications with systemic absorption, and androgens. Acute HAE attacks during the study were managed in accordance with the investigator’s usual practice, including use of individualized on-demand therapies. The on-demand therapies included icatibant, fresh frozen plasma, or other local standard of care treatment. Use of C1INH replacement therapy was permitted as an acute attack therapy but not as an LTP.

### 2.2 Patients

Inclusion and exclusion criteria are described in Supplementary Methods. For key inclusion criteria, patients were of Chinese descent and aged ≥12 years with a documented HAE-C1INH-Type1/2 diagnosis based on all of the following: clinical history consistent with HAE; confirmatory diagnostic test results obtained during screening (C1INH function <40% of normal or 40%–50% if C4 below normal range); and ≥1 of the following: aged ≤30 years at the onset of first angioedema symptoms and/or had a family history consistent with HAE-C1INH-Type1/2 or complement C1q within normal range. To be eligible for the study, patients must have experienced ≥1 investigator-confirmed HAE attack within 4 weeks during the run-in period.

Key exclusion criteria included concomitant diagnosis of another form of chronic recurrent angioedema (eg, acquired angioedema, HAE with normal C1INH, idiopathic angioedema), participation in a prior lanadelumab study or any use of lanadelumab before the study, exposure to ACE inhibitors or any estrogen-containing medications with systemic absorption ≤4 weeks prior to screening, exposure to androgens within ≤2 weeks prior to the run-in period, and use of short-term prophylaxis for HAE within ≤7 days prior to the run-in period.

### 2.3 Study endpoints and assessments

The primary objective was to evaluate the safety of repeated subcutaneous lanadelumab administration in Chinese patients with HAE based on treatment-emergent adverse events (TEAEs; coded using Medical Dictionary for Regulatory Activities Version 26.0), including AEs of special interest: hypersensitivity reactions and events of disordered coagulation. AEs were evaluated in 2 periods: treatment (days 0–182) and follow-up (after day 182) periods. Safety assessment details are reported in the Supplementary Methods.

Secondary objectives included evaluating the efficacy, pharmacokinetics, pharmacodynamics, and immunogenicity of lanadelumab. Efficacy was assessed in 2 evaluation periods: the overall treatment period (days 0–182) and the presumed lanadelumab steady-state period (days 70–182). Assessed outcomes included the number of investigator-confirmed HAE attacks; those that were treated and those deemed moderate or severe; maximum attack severity; time to first HAE attack; number and proportion of patients who were attack-free; number and percentage of patients meeting the criteria of ≥50%, ≥70%, ≥90%, and 100% reductions in the investigator-confirmed normalized number of attacks (NNA) per 4 weeks relative to the run-in period NNA; and number and percentage of patients achieving NNA <1.0 per 4 weeks. The occurrence or absence of HAE attacks were recorded daily by patients or caregivers using a diary card (Supplementary Methods), which was checked by study investigators and documented in patients’ electronic case report forms. Pharmacokinetics were assessed by measuring plasma concentrations of lanadelumab. Pharmacodynamics were assessed by the measurement of plasma levels of cleaved high-molecular-weight kininogen (cHMWK), an endogenous substrate and biomarker of plasma kallikrein activity that has been used as a pharmacodynamic marker for several plasma kallikrein inhibitors, including lanadelumab (18, 19, 32, 33). Details of lanadelumab pharmacokinetics, pharmacodynamics, and immunogenicity assessments are reported in Supplementary Methods and in other studies (18, 19).

### 2.4 Statistical analysis

Analyses of safety and efficacy data were based on the “full analysis set” (FAS; defined as all patients who received ≥1 dose of lanadelumab); as this was not a hypothesis testing study, no statistical comparisons were performed. The totality of results across all efficacy endpoints were the measure of overall treatment benefit. Continuous and categorical efficacy endpoints, pharmacokinetics, pharmacodynamics, and immunogenicity data were summarized using descriptive statistics. Overall attack rates per month (28 days) were estimated using summary statistics. Time-to-event endpoints were summarized using Kaplan–Meier estimates. A detailed description of the statistical methods is provided in the Supplementary Methods.

## 3 Results

### 3.1 Patient and disease characteristics at baseline

**Figure 1:**
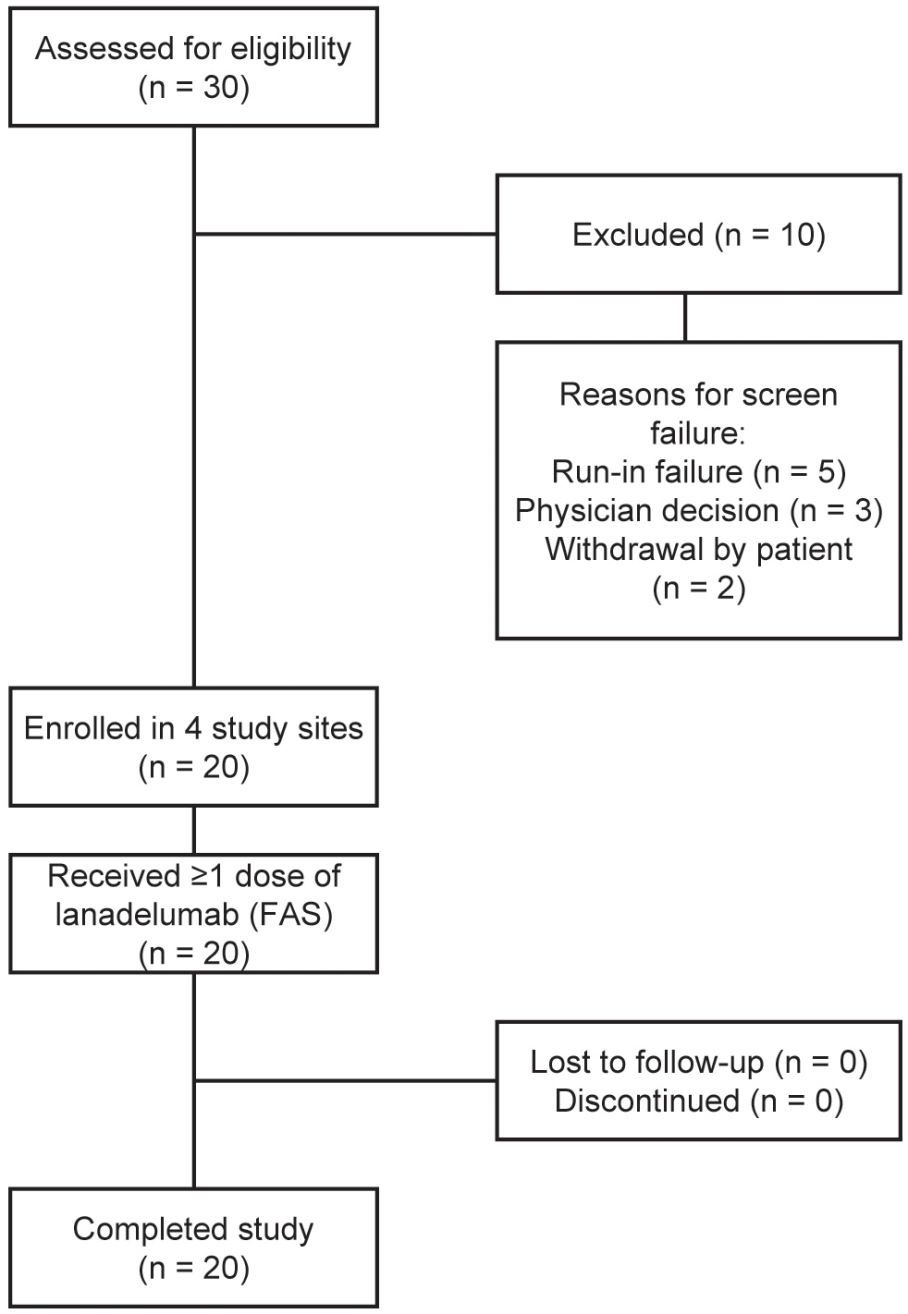
Patient flow diagram. *FAS*, full analysis set.

The study began on June 22, 2022, and ended on November 28, 2023. Twenty Chinese patients with confirmed HAE-C1INH diagnoses provided consent and were enrolled (**Figure 1**). Baseline and HAE attack characteristics are summarized in **Tables 1**, **2**, respectively. Medical history reported that 8 patients had HAE-C1INH-Type1, but HAE-C1INH was unspecified (Type1 or Type2) in the remainder. The median (range) monthly HAE attack rate during the run-in period was 1.93 (1.0–5.6). Notably, 70.0% of patients had a history of laryngeal attacks. Three patients (15.0%) reported prior history of LTP use (all androgens), which were discontinued >2 weeks before the run-in period.

**Table 1:** Patient baseline characteristics.

Characteristics	Total (N = 20)
Age at informed consent date	
Mean (SD), years	37.8 (11.60)
Median (range), years	38.0 (14–55)
<18 years, n (%)	1 (5.0)
18 to <40 years, n (%)	9 (45.0)
40 to <65 years, n (%)	10 (50.0)
Sex, n (%)	
Male	7 (35.0)
Female	13 (65.0)
BMI	
Mean (SD), kg/m^2^	24.18 (3.93)
Median (range), kg/m^2^	23.73 (18.6–31.3)
18.5 to <25.0 (normal)^a^, n (%)	11 (55.0)
25.0 to <30.0 (overweight)^a^, n (%)	5 (25.0)
≥30.0 (obese)^a^, n (%)	3 (15.0)
Healthy or overweight (5th to <95th percentile)^b^, n (%)	1 (5.0)
Age at onset of HAE symptoms, years	
Mean (SD)	16.5 (10.39)
Median (range)	16.0 (1–36)

^a^ Patients ≥18 years.

^b^ Patients <18 years. The BMI percentile categories were derived based on growth charts from the US Centers for Disease Control and Prevention.

BMI, body mass index; HAE, hereditary angioedema.

**Table 2:** Baseline HAE attack characteristics.

Characteristics	Total (N = 20)
History with laryngeal attack, n (%)	
Yes	14 (70.0)
No	6 (30.0)
Primary attack location, n (%)	
Laryngeal	0
Abdominal	1 (5.0)
Peripheral	1 (5.0)
Combination	18 (90.0)
HAE characteristics in the last 3 months prior to screening	
Number of attacks	
Mean (SD)	3.7 (2.3)
Median (range)	3.0 (0–8)
Number of attacks of different severity	
Mild	
Mean (SD)	2.6 (2.1)
Median (range)	2.5 (0–6)
Moderate	
Mean (SD)	0.9 (1.2)
Median (range)	0.0 (0–3)
Severe	
Mean (SD)	0.3 (0.6)
Median (range)	0.0 (0–2)
Average attack duration, n (%)	
<12 hours	0
12 to <24 hours	0
24 to <48 hours	4 (20.0)
≥48 hours	15 (75.0)
Not applicable	1 (5.0)
HAE characteristics during run-in period	
Attack rate, attacks/4 weeks	
Mean (SD)	2.5 (1.4)
Median (range)	1.9 (1.0–5.6)
Attack rate category, n (%)	
0 attacks/4 weeks	0
1 attack/4 weeks	12 (60.0)
2 attacks/4 weeks	2 (10.0)
≥3 attacks/4 weeks	6 (30.0)
LTP use before run-in period, n (%)	
No	17 (85.0)
Yes	3 (15.0)
C1INH	0
Androgens	3 (100)
Antifibrinolytics	0

C1INH, C1 inhibitor; HAE, hereditary angioedema; LTP, long-term prophylaxis.

### 3.2 Safety and tolerability

The median (range) duration of lanadelumab exposure during the study was 6.6 (6.5–6.9) months. Eighteen patients (90.0%) received all 14 doses of lanadelumab; 1 patient missed 1 dose because of COVID-19−related site restrictions, and another missed 1 dose due to a severe TEAE (described below).

Eighty-five non-HAE attack TEAEs occurred in 15 patients during the treatment period and 2 occurred in 2 patients during the follow-up period. None of these TEAEs were fatal or led to the discontinuation of lanadelumab treatment. No investigators reported AEs of special interest. The most common non-HAE attack TEAEs are shown in **Table 3**. During the follow-up period, 2 patients reported 2 non-serious, non-severe TEAEs of infections unrelated to lanadelumab treatment. There were no significant relationships between the incidence of non-HAE attack TEAEs and the run-in period HAE attack rate or LTP therapy use (**Supplementary Table 1**).

**Table 3:** Summary of non-HAE attack TEAEs.

TEAEs	Analysis period	
	Treatment period (days 0–182) (N = 20)	Follow-up period (day >182) (N = 20)
Any TEAE, number of patients (%) [number of events]	15 (75.0) [85]	2 (10.0) [2]
Treatment-related TEAE	9 (45.0) [41]	0
Serious TEAE	1 (5.0) [2]	0
Treatment-related serious TEAE	0	0
Severe TEAE	3 (15.0) [4]	0
Treatment-related severe TEAE	2 (10.0) [2]	0
Investigator-reported AESI	0	0
Fatal TEAE	0	0
Hospitalization due to TEAE	1 (5.0) [2]	0
Discontinuation due to TEAE	0	0
TEAEs occurring in ≥10% of patients		
COVID-19 infection	10 (50.0) [10]	0
Injection site pain	4 (20.0) [16]	0
Injection site swelling	3 (15.0) [7]	0
Musculoskeletal and connective tissue disorders	3 (15.0) [5]	0
Nausea	3 (15.0) [3]	0
Blood uric acid increased	2 (10.0) [3]	0
Skin and subcutaneous tissue disorders	2 (10.0) [3]	0
Eye disorders	2 (10.0) [2]	0
Abdominal pain upper	2 (10.0) [2]	0
Toothache	2 (10.0) [2]	0
Hepatic function abnormal	2 (10.0) [2]	0
Nasopharyngitis	2 (10.0) [2]	0
Dizziness	2 (10.0) [2]	0
Hypertension	2 (10.0) [2]	0
Treatment-related TEAEs occurring in ≥10% of patients		
Injection site pain	4 (20.0) [16]	0
Injection site swelling	3 (15.0) [7]	0
Hepatic function abnormal	2 (10.0) [2]	0

AESI, adverse event of special interest; HAE, hereditary angioedema; TEAE, treatment-emergent adverse event.

Nine patients experienced 41 TEAEs related to lanadelumab treatment; 30/41 were injection site reactions (ISRs) experienced by 5 patients (25.0%), most commonly injection site pain (20.0%) and/or injection site swelling (15.0%). All ISRs were mild and most (63.3%) lasted for ≤1 day. No serious treatment-related TEAEs were reported during the study period, but 2 severe TEAE events, considered by the investigator to be related to lanadelumab treatment, were reported in 2 patients: 1 experienced drug-induced liver injury (grade 3) and 1 experienced abnormal hepatic function (grade 4). The grade 3 TEAE of drug-induced liver injury occurred in a patient with no history of hepatic pathologies who had received androgens as LTP prior to enrollment; the TEAE resolved without changing lanadelumab dose. The grade 4 TEAE of abnormal hepatic function was reported in a patient with a history of abnormal hepatic function and chronic hepatitis B who was not on any LTP prior to enrollment; the TEAE led to the interruption of 1 dose of lanadelumab with the outcome of “not recovered.” It should be noted that 5 patients in the FAS had a history of hepatic pathologies, 4 of whom did not experience any treatment-related hepatic TEAEs. One patient experienced 2 severe and serious TEAEs of intervertebral disc protrusion and spinal stenosis (both grade 3) that were not related to lanadelumab treatment.

### 3.3 Efficacy

**Figure 2:**
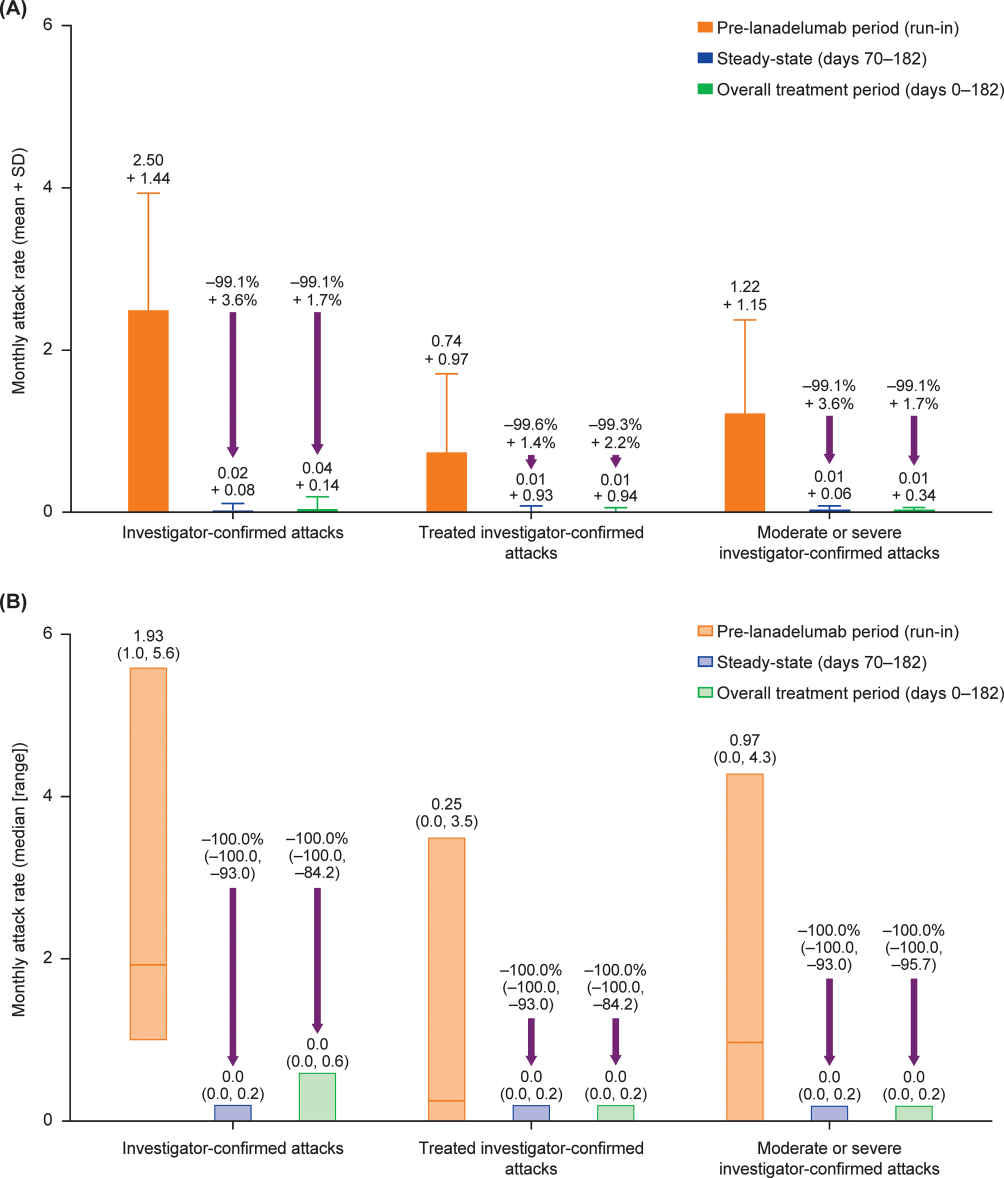
**(A)** Mean + SD and **(B)** median (range) investigator-confirmed HAE attack rate during the pre-lanadelumab run-in period, during the presumed lanadelumab steady-state period (days 70–182), and during the overall study period (days 0–182). Numbers above the purple arrows indicate mean + SD and median (range) percentage change in attack rate from the pre-lanadelumab run-in period in panels **(A)** and (**B**), respectively. All patients received lanadelumab 300 mg every 2 weeks. HAE, hereditary angioedema.

During the run-in period, the median (range) monthly attack rate was 1.93 (1.0–5.6); a total of 47 attacks were reported in the 20 patients, of which 14 (29.8%) and 21 (44.7%) attacks were treated with on-demand therapy and were considered moderate/severe, respectively. Following initiation of lanadelumab treatment, mean monthly rates of investigator-confirmed attacks, treated attacks, and attacks of moderate to severe severity all markedly decreased versus the run-in period (**Table 4**, **Figure 2**). The mean ± SD decreases from run-in in the monthly rate of investigator-confirmed attacks, treated attacks, and moderate/severe attacks during the treatment period were 99.1 ± 3.6%, 99.6 ± 1.4%, and 99.7 ± 1.1%, respectively.

**Table 4:** Summary of investigator-confirmed HAE attacks.

HAE attacks	All investigator-confirmed attacks			Attacks treated with on-demand therapy			Moderate to severe attacks		
	Run-in	Days 0–182 (N = 20)	Days 70–182 (N = 20)	Run-in	Days 0–182 (N = 20)	Days 70–182 (N = 20)	Run-in	Days 0–182 (N = 20)	Days 70–182 (N = 20)
Total patient-time, months	20.2​	131.8​	81.8​	20.2​	131.8​	81.8​	20.2​	131.8​	81.8​
Total number of attacks​, n	47​	5​	2​	14​	1​	1​	21​	1​	1​
Attack rate per month									
Mean (SD)​	2.50 (1.436)​	0.04 (0.139)​	0.02 (0.076)​	0.74 (0.966)​	0.01 (0.034)​	0.01 (0.055)​	1.22 (1.151)​	0.01 (0.034)​	0.01 (0.055)​
Median (range)​	1.93 (1.0–5.6​)	0.0 (0.0–0.6​)	0.0 (0.0–0.2​)	0.25 (0.0–3.5​)	0.00 (0.0–0.2​)	0.00 (0.0–0.2​)	0.97 (0.0–4.3​)	0.00 (0.0–0.2​)	0.00 (0.0–0.2​)
Change from run-in attack rate per month									
Mean (SD)	-	−2.46 (1.396)​	−2.47 (1.400)​	-	−0.73 (0.944)​	−0.73 (0.930)​	-	−1.21 (1.135)​	−1.20 (1.126)​
Median (range)	-	−1.93 (−5.6 to −1.0​)	−1.93 (−5.6 to −1.0​)	-	−0.25 (−3.3 to 0.0​)	−0.25 (−3.3 to 0.0​)	-	−0.97 (−4.3 to 0.0​)	−0.97 (−4.3 to 0.0​)
Percent change from run-in attack rate per month									
Mean (SD)	-	−99.07 (3.550)​	−99.45 (1.720)​	-	−99.57 (1.375)​	−99.30 (2.219)​	-	−99.71 (1.123)​	−99.53 (1.812)​
Median (range)	-	−100.00 (−100.0 to −84.2​)	−100.00 (−100.0 to −93.6​)	-	−100.00 (−100.0 to −95.7​)	−100.00 (−100.0 to −93.0​)	-	−100.00 (−100.0 to −95.7​)	−100.00 (−100.0 to −93.0​)

HAE, hereditary angioedema.

**Figure 3:**
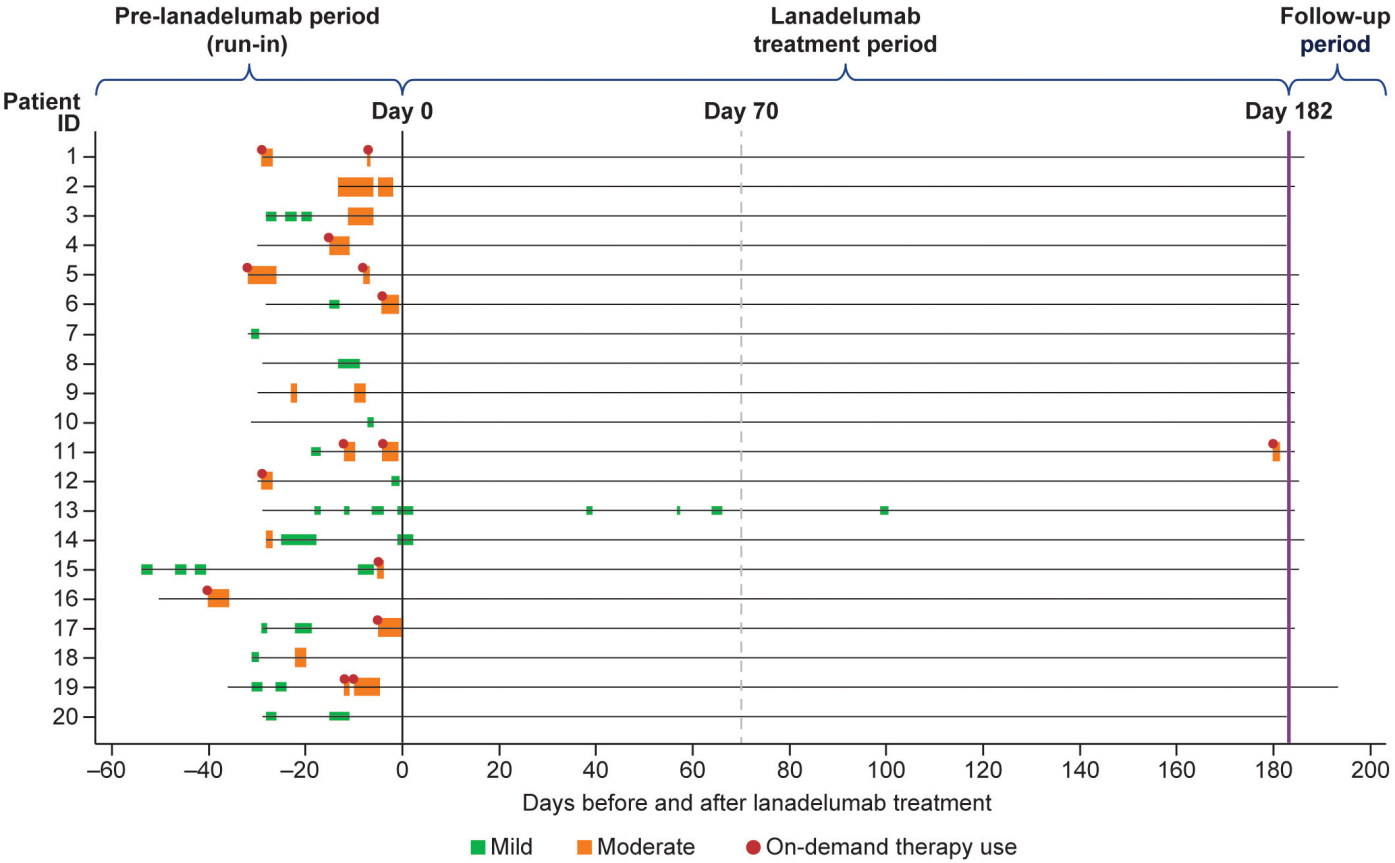
Investigator-confirmed HAE attacks during the run-in period and days 0–182. Each horizontal line represents data for an individual patient. The width of the boxes indicates the duration of the attack, and the color indicates the severity of the attack. Red circles indicate the use of on-demand medication. The solid line at day 0 represents the end of the run-in period. The dashed line at day 70 represents the start of steady-state period (days 70–182). The solid purple line at day 182 represents the end of the study period. All patients received lanadelumab 300 mg every 2 weeks. HAE, hereditary angioedema.

Over the treatment period, 18 patients (90.0%) were attack-free and 2 patients experienced 5 investigator-confirmed HAE attacks (primary attack locations were abdominal, n = 2; peripheral, n = 2; and laryngeal, n = 1). One patient experienced 4 mild attacks on days 39, 57, 66, and 100 and 1 patient experienced 1 moderate attack on day 181. Of the 2 investigator-confirmed HAE attacks during days 70–182, 1 was mild and the patient received no on-demand therapy and 1 was moderate and treated with icatibant (**Figure 3**): the mild attack lasted 37.0 hours and the moderate lasted 27.7 hours. The other 3 investigator-confirmed HAE attacks that occurred during the treatment period were mild and patients did not receive on-demand therapy. The results of the sensitivity analyses of patient-reported HAE attacks based on the FAS (**Supplementary Table 2**) were similar to the primary analyses of investigator-reported HAE attacks.

All patients achieved ≥70% reduction in the investigator-confirmed NNA/4 weeks from the run-in period; for days 70–182, all patients achieved a ≥90% reduction; for days 0–182, 95% of patients achieved a ≥90% reduction. Notably, 90% of patients achieved a 100% reduction in the investigator-confirmed NNA/4 weeks from the run-in period and all patients achieved the investigator-confirmed NNA <1.0/4 weeks during the efficacy period.

The mean ± SD percentage of attack-free days for patients during days 0–182 and 70–182 were 99.8 ± 0.62% and 99.9 ± 0.37%, respectively. The characteristics of investigator-confirmed and patient-reported HAE attacks during the treatment period are reported in **Table 5** and **Supplementary Table 3**, respectively. Kaplan–Meier estimates for the time to first investigator-confirmed HAE attack during the efficacy evaluation could not be estimated.

**Table 5:** Characteristics of investigator-confirmed HAE attacks by efficacy evaluation period.

Attack characteristics	Run-in (N = 20)	Days 0–182 (N = 20)	Days 70–182 (N = 20)
HAE attack duration for patients with HAE attacks, h			
n	20	2	2
Mean (SD)	65.54 (27.81)	29.01 (1.86)	32.35 (6.58)
Median (range)	61.24 (25.50–121.00)	29.01 (27.70–30.33)	32.35 (27.70–37.00)
Mean HAE attack duration category, n (%)			
No attack	0	18 (90.0)	18 (90.0)
<12 hours	0	0	0
12 to <24 hours	0	0	0
24 to <48 hours	6 (30.0)	2 (10.0)	2 (10.0)
≥48 hours	14 (70.0)	0	0
Maximum HAE attack severity, n (%)			
No attack	0	18 (90.0)	18 (90.0)
Mild	5 (25.0)	1 (5.0)	1 (5.0)
Moderate	15 (75.0)	1 (5.0)	1 (5.0)
Severe	0	0	0
Mean HAE attack severity for patients with HAE attacks^a^			
n	20	2	2
Mean (SD)	1.49 (0.40)	1.50 (0.71)	1.50 (0.71)
Median (range)	1.50 (1.00–2.00)	1.50 (1.00–2.00)	1.50 (1.00–2.00)

^a^ The mean attack severity was calculated using a numerical rating (0 = no attack, 1 = mild, 2 = moderate, and 3 = severe) and summarized for all patients, as well as only patients with HAE attacks.

HAE, hereditary angioedema.

### 3.4 Pharmacodynamics and pharmacokinetics

Upon commencing lanadelumab 300 mg q2w, the mean observed predose lanadelumab concentrations increased steadily during days 14–56, after which comparable concentrations were observed at all visits through day 182, suggesting the steady-state exposure to lanadelumab was achieved around day 56 (**Supplementary Figure 1**). The mean observed levels of cHMWK, a pharmacodynamic marker of lanadelumab treatment, showed a sustainable reduction of >50% after starting lanadelumab 300 mg q2w (**Supplementary Figure 2**). Notably, at the follow-up visit on day 210 (28 days after the last dose of lanadelumab was administered on day 182), the mean observed plasma lanadelumab concentration had decreased by approximately 50% but cHMWK levels remained suppressed.

### 3.5 Immunogenicity

At baseline, 1 patient (5.0%) was positive for antidrug antibodies (ADAs), without neutralizing ADAs (nADAs), but was ADA negative thereafter. Of the 19 patients (95.0%) who were ADA negative at baseline, 3 were positive for low-titer (range 60–240) treatment-emergent ADAs and for nADAs following lanadelumab initiation; 1 was positive for ADAs and nADAs from day 126 to the follow-up visit and 2 were positive for ADAs and nADAs at the follow-up visit. There were no investigator-confirmed attacks following lanadelumab initiation in patients with ≥1 positive ADA result, and no obvious differences in plasma lanadelumab concentrations or cHMWK levels versus ADA-negative patients (**Supplementary Figure 3**).

## 4 Discussion

Lanadelumab is approved for LTP of HAE in >60 countries and is currently the only approved LTP in China with first-line recommendation by the recently updated Chinese expert consensus (10, 12, 16). To our knowledge, this study is the first report of prospective clinical trial data for an LTP treatment in a study population representative of Chinese patients with HAE. Mean age of HAE symptom onset for patients in this study was 16.5 years, comparable with that of 17.5 years reported from a 2020 survey of 103 Chinese patients with HAE (9). Study results support the benefits of lanadelumab for LTP in Chinese patients with HAE-C1INH. These include substantial reductions in HAE attack rate from run-in following lanadelumab initiation and a sustained attack-free period for 90% of patients while receiving lanadelumab treatment. Moreover, we observed that Chinese patients with HAE presented similar pharmacokinetics and pharmacodynamics to patients from other clinical studies following repeated lanadelumab 300 mg q2w dosing (24). We showed that the safety profile of lanadelumab LTP in Chinese patients was consistent with that established in previous clinical studies (17–20). Among the 20 enrolled Chinese patients who received lanadelumab q2w treatment for a median of 6.57 months and completed the study, no TEAEs led to lanadelumab discontinuation, and none were fatal or were of special interest. Importantly, no new safety signals for lanadelumab were identified in this study population.

Nine patients (45.0%) had treatment-related AEs, most of which were mild to moderate ISRs reported in 5 patients (25.0%). The proportion of patients with treatment-related TEAEs in the current study was lower than reported in the HELP Study (59.5% across all lanadelumab arms) and a phase 3 study in Japanese patients (66.7%) (19, 20). The finding that ISRs were the most commonly reported treatment-related TEAEs is consistent with other lanadelumab clinical studies (19, 20), as well as 2 real-world studies in Chinese patients receiving lanadelumab for HAE LTP (30, 31). Of note, 2 patients (10%) experienced severe treatment-related hepatic TEAEs during the study period; 1 was using androgens for HAE LTP prior to enrollment and 1 had a history of chronic abnormal hepatic function and hepatitis B. Androgen use in patients with HAE has been associated with hepatotoxicity (34, 35). In the pivotal phase 3 HELP Study, 1 patient (1.2%) with a history of fatty liver experienced elevated hepatic enzymes that led to discontinuation of lanadelumab treatment (19). Although it is plausible that preexisting liver abnormalities could predispose selected patients to develop hepatic dysfunction, it should be noted that, in this study, 4 of the 5 patients with a history of hepatic pathologies and 2 of the 3 patients who were using androgens prior to enrollment experienced no treatment-related hepatic TEAEs.

Consistent with other lanadelumab clinical studies (19, 20), efficacy of lanadelumab as HAE LTP in Chinese patients was demonstrated by the reduced number and maximum severity of investigator-confirmed HAE attacks during lanadelumab treatment relative to the run-in period; the mean investigator-confirmed HAE attack rate during treatment days 0–182 decreased by 99% from the run-in period. The magnitude of attack-rate reduction observed in this study was numerically higher than the 77% reduction from run-in reported in a similar 26-week phase 3 study in Japanese patients with HAE (20) and the reduction in mean HAE attack rate versus placebo over 182 days in the HELP Study (87% in patients receiving lanadelumab 300 mg q2w) (19). The percentage of investigator-confirmed HAE attack-free patients during days 0–182 was also numerically higher in the current study (90.0%) than the 50% observed in the Japanese study and 44% observed in the HELP Study 300 mg q2w cohort (19, 20). Consistent with this, the percentage of patients meeting the criteria of ≥50%, ≥70%, and ≥90% reductions in the investigator-confirmed NNA/4 weeks versus the run-in period were also numerically higher than reported in the Japanese and HELP studies (19, 20). Importantly, our findings are also consistent with a recently published multicenter real-world prospective and retrospective study of 50 Chinese patients treated with lanadelumab, which included some patients from Peking Union Medical College and Guangzhou Medical University. The proportion of patients who were attack-free increased substantially, rising from 0% in the 6 months before starting lanadelumab treatment to 80.0% during the first 6 months of treatment in the study (31), consistent with other smaller real-world studies in China (patient number ranged between 1 and 11) that similarly showed lanadelumab was effective in preventing HAE attacks (27–30).

Lanadelumab plasma exposure and cHMWK levels observed in this study were comparable with those reported in patients from other clinical studies (18, 24). The population pharmacokinetics and pharmacodynamics of lanadelumab were characterized in a previous study using plasma samples collected from patients across different lanadelumab clinical trials (24). The lanadelumab 300 mg q2w regimen was found to result in a mean steady-state minimum plasma lanadelumab concentration that was approximately 4.5-fold higher than the half-maximal inhibitory concentration (IC_50_) of plasma kallikrein, and the lanadelumab plasma concentration was expected to exceed IC_50_ after approximately 15 hours following the first dose of lanadelumab 300 mg (24). As the pharmacokinetic and pharmacodynamic profiles of lanadelumab in Chinese patients were similar to other clinical study populations, it is expected that plasma kallikrein inhibition and corresponding clinical benefit of attack prevention would occur in a similarly rapid and sustained manner in Chinese patients.

Of the 3 patients who were ADA positive during the study, none experienced any investigator-confirmed HAE attacks during the study. Although no direct relationship could be assumed between immunogenicity and efficacy in this study, findings from other clinical studies indicate that the development of ADA and nADA against lanadelumab did not appear to adversely affect clinical response (14). Consistent with a previous report (18), we found no evidence of ADA status affecting lanadelumab pharmacokinetics or pharmacodynamics in Chinese patients.

In line with lanadelumab product labeling in the United States (14), the Chinese product label, as well as the recently updated Chinese expert consensus, recommend considering treatment interval extension from q2w to every 4 weeks (q4w) if adequate disease control has been achieved (ie, attack-free for >6 months) (10, 12, 16). Although this study only evaluated the q2w regimen because of the 26-week lanadelumab treatment period, outcomes from other studies indicate extended dosing interval may be suitable for Chinese patients who have achieved adequate disease control with lanadelumab. Results from the retrospective INTEGRATED Study of 198 European patients with HAE treated with lanadelumab showed that attack-free rates during follow-up were comparable for patients receiving q2w and q4w regimens (22). In a 2025 single-center retrospective study of Chinese patients treated with lanadelumab for HAE LTP, of 9 patients who received lanadelumab for more than 70 days, dosing intervals were extended from q2w to q4w in 5 patients and attack rates remained low (30). In another study of 50 Chinese patients, the proportion of patients who were free from attacks remained high with dosing-interval extension in 80% of patients during a median follow-up of 17.5 months (31). Altogether, findings from these real-world studies support individualized dosing of lanadelumab in Chinese patients.

As emphasized in the latest Chinese expert consensus, the overarching goal of LTP is to achieve complete disease control and normalize patients’ lives. The decision to start LTP should take into consideration factors including disease activity and severity, accessibility of on-demand treatment, and preferences and the financial circumstances of individual patients (10, 12). The present findings that lanadelumab treatment may offer rapid and sustained disease control in Chinese patients with HAE by reducing attack frequency and severity are important, as there is evidence of unmet treatment need. Until the recent approval of lanadelumab, attenuated androgens (recommended as second-line LTP by the updated Chinese expert consensus and World Allergy Organization/European Academy of Allergy and Clinical Immunology guidelines for HAE management) (1, 10, 12) and antifibrinolytics were the only available LTP options for patients in China (2, 36).

Findings from a 2020 survey suggested that among patients with HAE in China who were not receiving any prophylactic treatment for their disease, HAE often worsened at ages 20–29 years, after which time a slight respite could occur, although symptoms generally remained relatively severe until approximately age 50 years (9). Additionally, a 2019 study found that patients with HAE living in China reported significantly lower health-related quality of life scores than the general population, with unsatisfactory disease control cited as a risk factor for decreased physical component scores in the standardized patient-reported Short-Form 36 Health survey, which indicates perceptions of lower levels of physical health and functioning by the patient (13). Clinical trials (17, 19, 20) and real-world studies (37) have consistently demonstrated improved health-related quality of life and perception of disease control in lanadelumab-treated patients with HAE. Although patient-reported outcomes were not evaluated in this study, the quality of life benefits from lanadelumab treatment are supported by findings in 2 recently published real-world studies of Chinese patients receiving lanadelumab for HAE LTP, which observed marked improvements in patients’ perception of disease control and health-related quality of life from baseline following lanadelumab initiation (30, 31).

Measures broadening access to HAE family screening programs, such as the CaSE-HAE program in Hong Kong, have been shown to improve patients’ quality of life and reduce HAE-related costs (38). Recent initiatives aimed at fostering multidisciplinary collaboration, facilitating cross-regional referrals, and encouraging post-graduate education in rare diseases will also likely improve disease awareness among physicians and reduce diagnostic inertia (7). In addition, there are innovative strategies to leverage smartphone technologies to improve patients’ access to specialist physicians, who tend to concentrate in major cities (7). Together with the availability of effective LTP therapies, such as lanadelumab, these form the key pillars of improving outcomes of patients with HAE in China.

The small number of patients recruited across 4 study sites limits the generalizability of findings to the patient population with HAE in China. This study is also limited by the open-label study design, lack of a control group, and short follow-up. Study eligibility, determined during screening, required patients to have C1INH function <40% of normal or 40%–50% if C4 was below the normal range. The protocol did not require the necessary tests to differentiate between HAE-C1INH-Type1 and HAE-C1INH-Type2, as patients with either were eligible to enroll into the study. Where available, this information was extracted from the patient medical history, but consistent with the reported challenges in timely HAE diagnosis surrounding disease awareness and access to diagnostic capabilities in China (2, 13), HAE-C1INH subtype was documented in only 40% of the 20 patients enrolled. This observation is unlikely to be unique to China, as HAE-C1INH subtype was also unknown in 50% of patients enrolled in the Japanese lanadelumab phase 3 study (20).

Nonetheless, results showed that patients treated with lanadelumab 300 mg q2w for 26 weeks reported substantial reductions in investigator-confirmed HAE attack rates versus the run-in period. All 20 enrolled patients who received lanadelumab treatment completed the study with no new safety signals identified in this population. Most (95%) treatment-related AEs were of mild to moderate severity; 90% of patients achieved prolonged attack-free status, and a high percentage achieved threshold (≥50%, ≥70%, ≥90%) reductions in HAE attack rates versus the period before lanadelumab initiation. The safety and efficacy results presented in this prospective study are in line with those reported in previous lanadelumab studies and support the benefit of lanadelumab LTP for patients in China with HAE.

## Data Availability

The dataset, including the redacted study protocol, redacted statistical analysis plan, and individual participants' data supporting the results reported in this article, will be made available within 3 months from initial request to researchers who provide a methodologically sound proposal. The data will be provided after its de-identification, in compliance with applicable privacy laws, data protection, and requirements for consent and anonymization.

## References

[1] Maurer M Magerl M Betschel S Aberer W Ansotegui IJ Aygören-Pürsün E . The international WAO/EAACI guideline for the management of hereditary angioedema-the 2021 revision and update. Allergy. (2022) 77:1961–90. doi: 10.1111/all.15214. PMID: 35006617

[2] Honda D Li PH Jindal AK Katelaris CH Zhi YX Thong BY . Uncovering the true burden of hereditary angioedema due to C1-inhibitor deficiency: a focus on the Asia-Pacific region. J Allergy Clin Immunol. (2024) 153:42–54. doi: 10.1016/j.jaci.2023.09.039. PMID: 37898409

[3] Drouet C López-Lera A Ghannam A López-Trascasa M Cichon S Ponard D . SERPING1 variants and C1-INH biological function: a close relationship with C1-INH-HAE. Front Allergy. (2022) 3:835503. doi: 10.3389/falgy.2022.835503. PMID: 35958943 PMC9361472

[4] Li PH Pawankar R Thong BY Fok JS Chantaphakul H Hide M . Epidemiology, management, and treatment access of hereditary angioedema in the Asia Pacific region: outcomes from an international survey. J Allergy Clin Immunol Pract. (2023) 11:1253–60. doi: 10.1016/j.jaip.2022.12.021. PMID: 36584968

[5] Guan X Sheng Y Liu S He M Chen T Zhi Y . Epidemiology, economic, and humanistic burden of hereditary angioedema: a systematic review. Orphanet J Rare Dis. (2024) 19:256. doi: 10.1186/s13023-024-03265-z. PMID: 38978028 PMC11229247

[6] Cui Q Xu Q Yang Y Li W Huang N Chen H . The prevalence of hereditary angioedema in a Chinese cohort with decreased complement 4 levels. World Allergy Organ J. (2022) 15:100620. doi: 10.1016/j.waojou.2021.100620. PMID: 34992711 PMC8693024

[7] Li PH Huang JX Wang CT Wong JCY Chen H Chai B . Hereditary angioedema (HAE) in China: advancing awareness, access, advocacy and alliances from the Greater Bay Area to the global HAE community. Clin Exp Allergy. (2025) 55:659–70. doi: 10.1111/cea.70014. PMID: 40038863 PMC12325137

[8] Xu YY Jiang Y Zhi YX Yin J Wang LL Wen LP . Clinical features of hereditary angioedema in Chinese patients: new findings and differences from other populations. Eur J Dermatol. (2013) 23:500–4. doi: 10.1684/ejd.2013.2105. PMID: 24001409

[9] Cao Y Liu S Zhi Y . The natural course of hereditary angioedema in a Chinese cohort. Orphanet J Rare Dis. (2020) 15:257. doi: 10.1186/s13023-020-01526-1. PMID: 32962702 PMC7510061

[10] Zhi Y Xu Y Liu S Wang X . Chinese expert consensus on the diagnosis and treatment of hereditary angioedema (2024 edition). Chin J Allergy Clin Immunol. (2025) 19:1–10. doi: 10.3969/j.issn.1673-8705.2025.01.001. PMID: 40209692

[11] Busse PJ Christiansen SC Riedl MA Banerji A Bernstein JA Castaldo AJ . US HAEA Medical Advisory Board 2020 guidelines for the management of hereditary angioedema. J Allergy Clin Immunol Pract. (2021) 9:132–50.e3. doi: 10.1016/j.jaip.2020.08.046. PMID: 32898710

[12] Xu Y Liu S Wang X Chen W Cheng L Guo Y . Expert consensus on the diagnosis and treatment of hereditary angioedema in China (2024 Edition). Int Arch Allergy Immunol. (2026) 187:61–73. doi: 10.1159/000545808. PMID: 40209692

[13] Liu S Wang X Xu Y Xu Q Zhi Y . Health-related quality of life and its risk factors in Chinese hereditary angioedema patients. Orphanet J Rare Dis. (2019) 14:191. doi: 10.1186/s13023-019-1159-5. PMID: 31395105 PMC6686410

[14] U.S. Food and Drug Administration Takeda Pharmaceutical Company Limited . Highlights of prescribing information. Takhzyro™ (lanadelumab-flyo) injection, for subcutaneous use (2025). Available online at: https://www.accessdata.fda.gov/drugsatfda_docs/label/2023/761090s010lbl.pdf (Accessed March 19, 2026).

[15] European Medicines Agency Takeda Pharmaceutical Company Limited . TAKHZYRO® (lanadelumab-flyo) summary of product characteristics (2025). Available online at: https://www.ema.europa.eu/en/documents/product-information/takhzyro-epar-product-information_en.pdf (Accessed March 19, 2026).

[16] Takeda . 拉那利尤单抗注射液说明书 (lanadelumab manual) (2025). Available online at: https://assets-dam.takeda.com/image/upload/v1750657309/legacy-dotcom/siteassets/zh-cn/home/what-we-do/our-products/达泽优-拉那利尤单抗注射液-说明书-20250620-PFS.pdf (Accessed March 19, 2026).

[17] Banerji A Bernstein JA Johnston DT Lumry WR Magerl M Maurer M . Long-term prevention of hereditary angioedema attacks with lanadelumab: the HELP OLE Study. Allergy. (2022) 77:979–90. doi: 10.1111/all.15011. PMID: 34287942 PMC9292251

[18] Banerji A Busse P Shennak M Lumry W Davis-Lorton M Wedner HJ . Inhibiting plasma kallikrein for hereditary angioedema prophylaxis. N Engl J Med. (2017) 376:717–28. doi: 10.1056/NEJMoa1605767. PMID: 28225674

[19] Banerji A Riedl MA Bernstein JA Cicardi M Longhurst HJ Zuraw BL . Effect of lanadelumab compared with placebo on prevention of hereditary angioedema attacks: a randomized clinical trial. JAMA. (2018) 320:2108–21. doi: 10.1001/jama.2018.16773. PMID: 30480729 PMC6583584

[20] Hide M Ohsawa I Nurse C Yu M for the SHP643–302 Trial Investigators . Efficacy and safety of lanadelumab in Japanese patients with hereditary angioedema: a phase 3 multicenter, open-label study. J Dermatol. (2023) 50:1381–91. doi: 10.1111/1346-8138.16909. PMID: 37574953

[21] Bernstein JA Betschel SD Busse PJ Banerji A Wedner HJ Manning M . Sustained effectiveness, tolerability, and safety of long-term prophylaxis with lanadelumab in hereditary angioedema: the prospective, phase 4, noninterventional EMPOWER real-world study. Adv Ther. (2025) 42:3882–901. doi: 10.1007/s12325-025-03226-3. PMID: 40504359 PMC12313730

[22] Magerl M Bouillet L Martinez-Saguer I Gavini F Bent-Ennakhil N Sayegh L . Real-world effectiveness of lanadelumab in hereditary angioedema: multicountry INTEGRATED observational study. J Allergy Clin Immunol Pract. (2025) 13:378–87.e2. doi: 10.1016/j.jaip.2024.12.008. PMID: 39701274

[23] Tachdjian R Banerji A Busse PJ Agmon-Levin N Anderson J Cancian M . Effective long-term prophylaxis with lanadelumab in adolescents with hereditary angioedema: EMPOWER/ENABLE. Pediatr Allergy Immunol. (2025) 36:e70072. doi: 10.1111/pai.70072. PMID: 40171989 PMC11963219

[24] Wang Y Marier JF Kassir N Chang C Martin P . Pharmacokinetics, pharmacodynamics, and exposure-response of lanadelumab for hereditary angioedema. Clin Transl Sci. (2020) 13:1208–16. doi: 10.1111/cts.12806. PMID: 32407574 PMC7719386

[25] Sexton D Kichev A Juethner S Yeung D MacDonald A Anokian E . Hereditary angioedema plasma proteomics following specific plasma kallikrein inhibition with lanadelumab. Front Immunol. (2024) 15:1471168. doi: 10.3389/fimmu.2024.1471168. PMID: 40417315 PMC12098075

[26] Chyung Y Vince B Iarrobino R Sexton D Kenniston J Faucette R . A phase 1 study investigating DX-2930 in healthy subjects. Ann Allergy Asthma Immunol. (2014) 113:460–6.e2. doi: 10.1016/j.anai.2014.05.028. PMID: 24980392

[27] Du W Yang K Zhang Q Lin X Zhang W Guo W . Case report: Identification of a novel mutation, c.1067T > A, in the SERPING1 gene in a Chinese male with type 1 hereditary angioedema. Front Allergy. (2025) 6:1554940. doi: 10.3389/falgy.2025.1554940. PMID: 40364801 PMC12069465

[28] Wong JCY Chiang V Lam DLY Lee E Lam K Au EYL . Long-term prophylaxis for hereditary angioedema: initial experiences with garadacimab and lanadelumab. J Allergy Clin Immunol Glob. (2023) 2:100166. doi: 10.1016/j.jacig.2023.100166. PMID: 38024849 PMC10679768

[29] Yao W Diao R Yang B Wang Y Li B Li T . Initial experience of long-term prophylaxis with lanadelumab for hereditary angioedema in China: a clinical observation study on six patients. Int Arch Allergy Immunol. (2025) 186:221–31. doi: 10.1159/000541242. PMID: 39362190

[30] Xu Y Guo Y . A single-centre retrospective study on the clinical characteristics of patients with hereditary angioedema and the therapeutic effect of lanadelumab. Orphanet J Rare Dis. (2025) 20:441. doi: 10.1186/s13023-025-03988-7. PMID: 40826407 PMC12362876

[31] Xu Y Ji R Liu J Cui X Zhang H Cao N . Efficacy and acceptability of lanadelumab for long-term prophylaxis in hereditary angioedema: A Chinese multicenter real-world analysis. World Allergy Organ J. (2026) 19:101165. doi: 10.1016/j.waojou.2025.101165. PMID: 41550678 PMC12805351

[32] Lumry W Gunsior M Cohen T Bernard K Gustafson P Chung JK . Safety and pharmacokinetics of long-acting plasma kallikrein inhibitor navenibart (STAR-0215) in healthy adults. Ann Allergy Asthma Immunol. (2025) 135:103–11.e2. doi: 10.1016/j.anai.2025.03.016. PMID: 40158724

[33] Maetzel A Smith MD Duckworth EJ Hampton SL De Donatis GM Murugesan N . KVD900, an oral on-demand treatment for hereditary angioedema: phase 1 study results. J Allergy Clin Immunol. (2022) 149:2034–42. doi: 10.1016/j.jaci.2021.10.038. PMID: 35086692

[34] Guo Y Zhang H Lai H Wang H Chong-Neto HJ Valle SOR . Long-term prophylaxis with androgens in the management of hereditary angioedema (HAE) in emerging countries. Orphanet J Rare Dis. (2022) 17:399. doi: 10.1186/s13023-022-02536-x. PMID: 36324138 PMC9632066

[35] Riedl MA . Critical appraisal of androgen use in hereditary angioedema: a systematic review. Ann Allergy Asthma Immunol. (2015) 114:281–8.e7. doi: 10.1016/j.anai.2015.01.003. PMID: 25707325

[36] Zhi Y An L Lai H Liu RL Sun YM Wen LP . Expert consensus on the diagnosis and treatment of hereditary angioedema. Chin J Allergy Clin Immunol. (2019) 13:1–4. 35900448

[37] Zanichelli A Wuillemin WA Aygören-Pürsün E Banerji A Busse PJ Betschel SD . Lanadelumab's impact on hereditary angioedema control and quality of life across disease activity subgroups: real-world evidence. Ann Allergy Asthma Immunol. (2025) 135:560−9.e2. doi: 10.1016/j.anai.2025.07.025. PMID: 40769455

[38] Wong JCY Chiang V Lam K Tung E Au EYL Lau CS . Prospective study on the efficacy and impact of Cascade Screening and Evaluation of Hereditary Angioedema (CaSE-HAE). J Allergy Clin Immunol Pract. (2022) 10:2896–903.e2. doi: 10.1016/j.jaip.2022.07.035. PMID: 35964924

