# Supplementary material for: Lanadelumab safety, efficacy, and pharmacokinetics in patients aged ≥12 with hereditary angioedema in China: an open-label, multicenter study
Source: Front Immunol. 2026 Apr 29;17:1750735. doi: 10.3389/fimmu.2026.1750735 (PMC13167535; doi:10.3389/fimmu.2026.1750735)

Lanadelumab Safety, Efficacy, and Pharmacokinetics in Patients Aged ≥12 With Hereditary Angioedema in China: An Open-Label, Multicenter Study

Yuxiang Zhi,[1]* Rongfei Zhu,[2] Yuemei Sun,[3] He Lai,[4] Hong Ren,[5] Yi Wang,[5] Lin Dong[6]

[1]Department of Allergy, Peking Union Medical College Hospital, Chinese Academy of Medical Sciences, Beijing, China
[2]Department of Allergy, Tongji Hospital, Tongji Medical College, Huazhong University of Science & Technology, Wuhan, China
[3]Department of Allergy, Yantai Yuhuangding Hospital, Yantai, Shandong, China
[4]Department of Allergy, The Second Affiliated Hospital of Guangzhou Medical University, Guangzhou, Guangdong, China
[5]Takeda Development Center Americas, Inc., Cambridge, MA, United States
[6]Takeda APAC Biopharmaceutical Research and Development Company Limited, Shanghai, China

*** Correspondence:**
Yuxiang Zhi, MD, PhD
yuxiang_zhi@126.com

Supplementary Material

1 Supplementary Methods

1.1 Inclusion and Exclusion Criteria

1.1.1 Inclusion Criteria

- The patient was of Chinese descent, defined as born in China and having Chinese parents and Chinese maternal and paternal grandparents.
- The patient was male or female and ≥12 years of age at the time of informed consent.
- Documented diagnosis of hereditary angioedema due to C1INH deficiency (HAE-C1INH-Type1/2) based upon all of the following:
  - Documented clinical history consistent with HAE (subcutaneous or mucosal, nonpruritic swelling episodes without accompanying urticaria).
  - Diagnostic testing results obtained during screening by a laboratory (approved by the sponsor) that confirm HAE-C1INH-Type1/2: C1INH functional level <40% of the normal level. Patients with functional C1INH level at 40% to 50% of the normal level may be enrolled if they also have a C4 level below the normal range. Patients could begin participating in the run-in period before these diagnostic results were available. Patients may be re-tested if results were incongruent with clinical history or believed by the investigator to be confounded by recent LTP use.
  - At least 1 of the following: age at reported onset of first angioedema symptoms ≤30 years, a family history consistent with HAE-C1INH-Type1/2, or complement C1q within normal range.
- At the time of enrollment, patients must have experienced ≥1 investigator-confirmed HAE attack per 4 weeks during the run-in period.

- The patient (or their parent/legal guardian, if applicable) provided written informed consent approved by the institutional review board or independent ethics committee.
    - If the patient was an adult, they were informed of the nature of the study and provided written informed consent before any study-specific procedures were performed.
    - If the patient was a minor (ie, <18 years of age), they had a parent/legal guardian who was informed of the nature of the study and provided written informed consent (ie, permission) for the minor to participate in the study before any study-specific procedures were performed. Assent was obtained from patients who were minors.
- Males, or nonpregnant, nonlactating females who were fertile and sexually active and who agreed to be abstinent or agreed to comply with the applicable contraceptive requirements for the duration of the study, or females of nonchildbearing potential, defined as surgically sterile (status post hysterectomy, bilateral oophorectomy, or bilateral tubal ligation) or postmenopausal for ≥12 months.
- The patients agreed to adhere to the protocol-defined schedule of assessments and procedures.

1.1.2 Exclusion Criteria

- Concomitant diagnosis of another form of chronic recurrent angioedema, such as acquired angioedema, HAE with normal C1INH, idiopathic angioedema, or recurrent angioedema associated with urticaria.
- Participation in a prior lanadelumab study or any use of lanadelumab prior to the study.
- Dosing with investigational drug or exposure to an investigational device within 4 weeks prior to screening.
- Exposure to angiotensin-converting enzyme (ACE) inhibitors or any estrogen-containing medications with systematic absorption (eg, oral contraceptives or hormonal replacement therapy) within 4 weeks prior to screening.
- Exposure to androgens (eg, danazol, methyltestosterone, testosterone) within 2 weeks prior to entering the run-in period.
- Use of LTP therapy (defined as continued use) for HAE (C1INH, attenuated androgens, or antifibrinolytics) for adult patients within 2 weeks prior to entering the run-in period. Adolescent patients (≥12 to <18 years of age) who were receiving LTP therapy for HAE were allowed to enter the study.
- Use of short-term prophylaxis for HAE (defined as fresh frozen plasma, C1INH, attenuated androgens, or antifibrinolytics used to avoid angioedema complications from medically indicated procedures) 7 days prior to entering the run-in period.
- Any of the following liver function abnormalities: alanine aminotransferase >3× upper limit of normal (ULN), or aspartate aminotransferase >3× ULN, or bilirubin >2× ULN (unless the bilirubin is a result of Gilbert's syndrome).
- Pregnant or breastfeeding.
- Any condition that, in the opinion of the investigator or sponsor, might compromise the patient's safety or compliance, preclude successful conduct of the study, or interfere with interpretation of the results (eg, history of substance abuse or dependence, significant preexisting illnesses or major comorbidity that the investigator considered could confound the interpretation of the study results).

1.2 Safety Assessments

An adverse event (AE) was deemed serious if it was an important medical event, required inpatient hospitalization or prolongation of existing hospitalization, resulted in persistent or significant disability/incapacity, resulted in a congenital abnormality/birth defect, was life-threatening, or resulted in death. All AEs/serious AEs (SAEs) were collected from the time the informed consent document was

signed until the defined follow-up period and visit 15 (day 210). This included events that occurred during the screening phase of the study, regardless of whether or not lanadelumab was administered.

If the patient experienced a change in the severity of an AE, the event was to be captured once, with the maximum severity recorded. However, worsening medical conditions, or signs or symptoms present prior to initiation of investigational product, were recorded as new AEs. Severities of AEs were assessed and graded according to DMID Adult Toxicity Table and DMID Pediatric Toxicity Table.

For abnormalities not specifically found in the toxicity tables, the following general scale was used to estimate severity grade:

- GRADE 1 (Mild): Transient or mild discomfort; no medical intervention/therapy required
- GRADE 2 (Moderate): Mild to moderate limitation in activity, some assistance may be needed; no or minimal medical intervention/therapy required
- GRADE 3 (Severe): Marked limitation in activity, some assistance usually required; medical intervention/therapy required, hospitalizations possible
- GRADE 4 (Life-threatening): Extreme limitation in activity, significant assistance required; significant medical intervention/therapy required, hospitalization or hospice care probable

AEs of special interest (AESIs) were captured and monitored during this study. Investigators reported all AESIs regardless of causality, using the same timelines as described for SAE reporting. The following described the AESIs and the criteria for reporting AESIs:

- Hypersensitivity reactions

As hypersensitivity reactions have been observed for monoclonal antibodies as a therapeutic class, these events were considered AESIs in this study. Investigators reported all diagnoses, or signs and symptoms when diagnoses could not be determined, that were consistent with hypersensitivity reactions, regardless of causality, within 24 hours from the time of study drug administration. Investigators reported hypersensitivity reactions that occurred after 24 hours, only if the reactions were suspected to be related to study drug.

- Events of disordered coagulation
    - Bleeding AESI

Although activated partial thromboplastin time (aPTT) prolongation due to plasma kallikrein inhibition was an artifactual in vitro phenomenon, as a precautionary measure in evaluating the safety of lanadelumab, bleeding events were reported as AESIs in this study. Investigators reported all diagnoses, or signs and symptoms when diagnoses cannot be determined, that were consistent with a clinical event of bleeding. Coagulation testing (aPTT, international normalized ratio, prothrombin time) was performed when possible, and when temporally reasonable, with any reports of bleeding or for clinical conditions possibly indicative of bleeding.

- Hypercoagulable AESI

Investigators reported all diagnoses, or signs and symptoms when diagnoses could not be determined, that were consistent with a thrombotic or embolic etiology.

Other safety endpoints included clinical laboratory testing, vital signs including blood pressure, heart rate, body temperature and respiratory rate, 12-lead electrocardiogram, and physical examination.

1.3 HAE Attack Diary Card

Patients or caregivers were asked to complete a diary card at the end of each day to record if an HAE attack had happened. An HAE attack worksheet (part of diary card) was completed by the patient or caregiver within 72 hours of the onset of the HAE attack, with the following information recorded: date and time when symptoms of an attack were first experienced; symptoms experienced and anatomical location(s); impact on activity and whether any assistance or medical intervention was required, including

hospitalizations or emergency department visits; any medications used to treat the attack; if the attack resolved; and date and time the patient was no longer experiencing symptoms. All patient-reported and investigator-confirmed HAE attacks were recorded in the electronic case report form (eCRF).

1.4 Pharmacokinetic, Pharmacodynamic, and Immunogenicity Assessments

Plasma samples were collected from patients on days 0, 14, 56, 98, 140, and 182 (end of treatment) within 2 hours before dosing, and day 210 (follow-up). Measurements of plasma concentrations of lanadelumab and cleaved high-molecular-weight kininogen (pharmacodynamic biomarker of plasma kallikrein) were performed by Q^2 Solutions Beijing (Beijing, China) using validated enzyme-linked immunosorbent assays. Plasma samples for antidrug antibody (ADA) analysis were collected on days, 0, 56, 98, 140, 182, and 210; measurements of ADA were performed by Q^2 Solutions Beijing (Beijing, China) using a validated method of electrochemiluminescence.

1.5 Statistical Analysis

1.5.1 Sample Size Determination and Analysis Populations

The planned total sample size calculated for the study was ~20 patients, corresponding to ~87% probability of observing an event that occurs within the population at a rate of 10%. Analysis of efficacy and safety data was based on the "full analysis set" (FAS; defined as all patients who received ≥1 dose of lanadelumab). All pharmacokinetic analyses were based on the "PK set," defined as all patients in the FAS with ≥1 evaluable postdose pharmacokinetic concentration value. All pharmacodynamic analyses were based on the "pharmacodynamic set," defined as all patients in the FAS with ≥1 evaluable postdose pharmacodynamic concentration value.

1.5.2 Efficacy Analyses

No statistical hypothesis testing was performed. The totality of results across all efficacy endpoints was the measure of overall treatment benefit. Efficacy endpoints were evaluated for the following 2 efficacy evaluation periods: days 0 (after study drug administration) through day 182 and the presumed steady-state period from day 70 through day 182. For efficacy evaluation periods starting from day 70, only patients who reached the visit of day 70 were included in the analysis, and this number of patients was used as the denominator for percentage calculation. An HAE attack was counted for a specific efficacy evaluation period only if that HAE attack started during that period (eg, if an HAE attack started before day 70 and is ongoing after day 70, it was not counted for the efficacy period day 70 through day 182).

Investigator-confirmed HAE attacks were those that the primary investigator confirmed as meeting the Hereditary Angioedema Attack Assessment and Reporting Procedures (HAARP) criteria for an HAE attack and reported in the eCRF. Investigator-confirmed HAE attacks treated with on-demand therapy were defined as those attacks with "Has the subject received any acute HAE therapy treatment for this reported attack?" marked as "Yes" in the eCRF. Moderate and severe investigator-confirmed HAE attacks were defined as those attacks that were classified as being moderate or severe according to the HAARP-defined severity and reported as such in the eCRF. The numbers of investigator-confirmed HAE attacks, attacks treated with on-demand therapy, and moderate or severe attacks during each efficacy evaluation period were expressed as a normalized monthly rate (4 weeks, ie, 28 days). The baseline investigator-confirmed HAE attack rate was calculated for each patient as the number of investigator-confirmed HAE attacks occurring during the baseline run-in period divided by the number of days the patient contributed to the run-in period multiplied by 28 days. The baseline investigator-confirmed attack rate, as well as the treatment period investigator-confirmed attack rate, change from baseline, and percent change from baseline for each efficacy evaluation period were summarized for the FAS. In addition to the descriptive statistics for attack rates, the summary included the total number of investigator-confirmed HAE attacks reported during each period and patient-time in months that each patient contributed to each period. The number and percentage of patients were also tabulated by maximum attack severity (no attacks, mild, moderate, and severe) for each efficacy evaluation period.

The time to the first investigator-confirmed HAE attack (days) after day 0 for the efficacy evaluation periods of days 0–182 and days 70–182 was calculated from the date and time of the first dose of lanadelumab for that efficacy evaluation period to the date and time of the first investigator-confirmed HAE attack after the first dose for that efficacy evaluation period. Time to the first investigator-confirmed HAE attack was summarized using Kaplan-Meier estimates of the 25th, 50th (median), and 75th percentiles, if estimable, and with associated 2-sided 95% CI, as well as percentage of events and censored observations.

Sensitivity analyses were performed to evaluate the robustness of the efficacy results during each efficacy evaluation period. The analysis was repeated using all patient-reported HAE attacks instead of investigator-confirmed HAE attacks.

2 Supplementary Tables

Supplementary Table 1. TEAEs during treatment period (days 0–182) as categorized by the run-in period HAE attack rate and prior LTP therapy use.

**Category, number of patients (%) [number of events]**	**By run-in period HAE attack rate**			**By LTP use prior to enrollment**		**All patients (N = 20)**
	**≤1 attacks/4 weeks (n = 12)**	**2 attacks/4 weeks (n = 2)**	**≥3 attacks/4 weeks (n = 6)**	**LTP use (n = 3)**	**No LTP use (n = 17)**	
TEAEs	8 (66.7) [54]	2 (100.0) [9]	5 (83.3) [22]	3 (100.0) [16]	12 (70.6) [69]	15 (75.0) [85]
Treatment-related TEAEs	5 (41.7) [29]	1 (50.0) [1]	3 (50.0) [11]	1 (33.3) [4]	8 (47.1) [37]	9 (45.0) [41]
Serious TEAEs	0	1 (50.0) [2]	0	0	1 (5.9) [2]	1 (5.0) [2]
Severe TEAEs	2 (16.7) [2]	1 (50.0) [2]	0	1 (33.3) [1]	2 (11.8) [3]	3 (15.0) [4]
TEAE of special interest	0	0	0	0	0	0
Fatal TEAEs	0	0	0	0	0	0
Hospitalization due to TEAE	0	1 (50.0) [2]	0	0	1 (5.9) [2]	1 (5.0) [2]

HAE, hereditary angioedema; LTP, long-term prophylaxis; TEAE, treatment-emergent adverse event.

Supplementary Table 2. Summary of patient-reported HAE attacks.

	Patient-reported attacks			Attacks treated with on-demand therapy			Moderate to severe attacks		
	**Run-in**	**Days 0–182 (N = 20)**	**Days 70–182 (N = 20)**	**Run-in**	**Days 0–182 (N = 20)**	**Days 70–182 (N = 20)**	**Run-in**	**Days 0–182 (N = 20)**	**Days 70–182 (N = 20)**
Total patient-time, months	20.2	131.8	81.8	20.2	131.8	81.8	20.2	131.8	81.8
Total number of attacks	47	6[a]	2	14	2[a]	1	21	2[a]	1
Attack rate per month									
Mean (SD)	2.50 (1.436)	0.05 (0.140)	0.02 (0.076)	0.74 (0.966)	0.02 (0.047)	0.01 (0.055)	1.22 (1.151)	0.02 (0.047)	0.01 (0.055)
Median (range)	1.93 (1.0–5.6)	0.0 (0.0–0.6)	0.0 (0.0–0.2)	0.25 (0.0–3.5)	0.00 (0.0–0.2)	0.00 (0.0–0.2)	0.97 (0.0–4.3)	0.00 (0.0–0.2)	0.00 (0.0–0.2)
Change from run-in attack rate per month									
Mean (SD)	-	−2.45 (1.400)	−2.47 (1.400)	-	−0.72 (0.942)	−0.73 (0.930)	-	−1.20 (1.137)	−1.20 (1.126)
Median (range)	-	−1.93 (−5.6 to −1.0)	−1.93 (−5.6 to −1.0)	-	−0.25 (−3.3 to 0.0)	−0.25 (−3.3 to 0.0)	-	−0.97 (−4.3 to 0.0)	−0.97 (−4.3 to 0.0)
Percent change from run-in attack rate per month									
Mean (SD)	-	−98.68 (3.860)	−99.45 (1.720)	-	−98.00 (4.995)	−99.30 (2.219)	-	−98.67 (4.122)	−99.53 (1.812)
Median (range)	-	−100.00 (−100.0 to −84.2)	−100.00 (−100.0 to −93.6)	-	−100.00 (−100.0 to −84.3)	−100.00 (−100.0 to −93.0)	-	−100.00 (−100.0 to −84.3)	−100.00 (−100.0 to −93.0)

[a]One patient-reported HAE attack was not investigator confirmed, which had the alternative diagnosis of probable COVID-19 infection, with a maximum attack severity of severe.
HAE, hereditary angioedema.

Supplementary Table 3. Characteristics of patient-reported HAE attacks by efficacy evaluation period.

	**Run-in (N = 20)**	**Days 0-182 (N = 20)**	**Days 70-182 (N = 20)**
HAE attack duration for all patients, hours			
Mean (SD)	65.54 (27.81)	4.94 (12.26)	3.24 (10.07)
Median (range)	61.24 (25.50–121.00)	0.00 (0.00–40.70)	0.00 (0.00–37.00)
HAE attack duration for patients with HAE attacks, hours			
n	20	3	2
Mean (SD)	65.54 (27.81)	32.91 (6.87)	32.35 (6.58)
Median (range)	61.24 (25.50–121.00)	30.33 (27.70–40.70)	32.35 (27.70–37.00)
Mean HAE attack duration category, n (%)			
No attack	0	17 (85.0)	18 (90.0)
<12 hours	0	0	0
12 to <24 hours	0	0	0
24 to <48 hours	6 (30.0)	3 (15.0)	2 (10.0)
≥48 hours	14 (70.0)	0	0
Maximum HAE attack severity, n (%)			
No attack	0	17 (85.0)	18 (90.0)
Mild	5 (25.0)	1 (5.0)	1 (5.0)
Moderate	15 (75.0)	1 (5.0)	1 (5.0)
Severe	0	1 (5.0)	0
Mean HAE attack severity for all patients			
n	20	20	20
Mean (SD)	1.49 (0.40)	0.30 (0.80)	0.15 (0.49)
Median (range)	1.50 (1.00–2.00)	0.00 (0.00–3.00)	0.00 (0.00–2.00)
Mean HAE attack severity for patients with HAE attacks			
n	20	3	2
Mean (SD)	1.49 (0.40)	2.00 (1.00)	1.50 (0.71)
Median (range)	1.50 (1.00–2.00)	2.00 (1.00–3.00)	1.50 (1.00–2.00)
Number of patients with events, n (%)	-	3 (15.0)	2 (10.0)
Number of patients censored, n (%)	-	17 (85.0)	18 (90.0)
Time to first HAE attack Kaplan-Meir Estimate, days (95% CI)			
25th	-	NE (37.1, NE)	NE (27.4, NE)
50th	-	NE	NE
75th	-	NE	NE
Percentage reduction of HAE attack rate relative to run-in period, n (%)			
≥50% reduction	-	20 (100.0)	20 (100.0)
≥70% reduction	-	20 (100.0)	20 (100.0)
≥90% reduction	-	19 (95.0)	20 (100.0)
100% reduction	-	17 (85.0)	18 (90.0)

HAE, hereditary angioedema; NE, not evaluated.

3 Supplementary Figures

Supplementary Figure 1. Mean (SD) plasma lanadelumab concentrations in Chinese patients with hereditary angioedema by study visit.

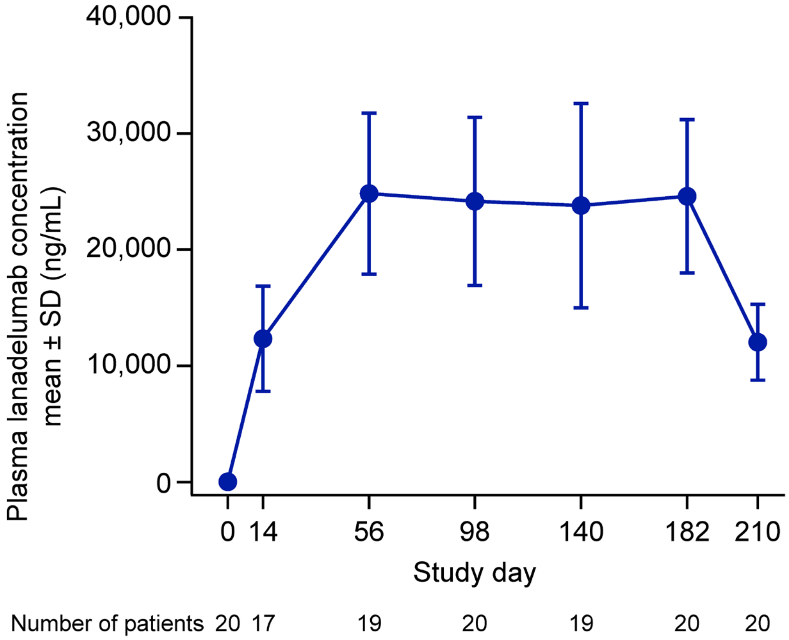

Supplementary Figure 2. Mean (SD) cHMWK levels in Chinese patients with hereditary angioedema by study visit. cHMWK, cleaved high-molecular-weight kininogen.

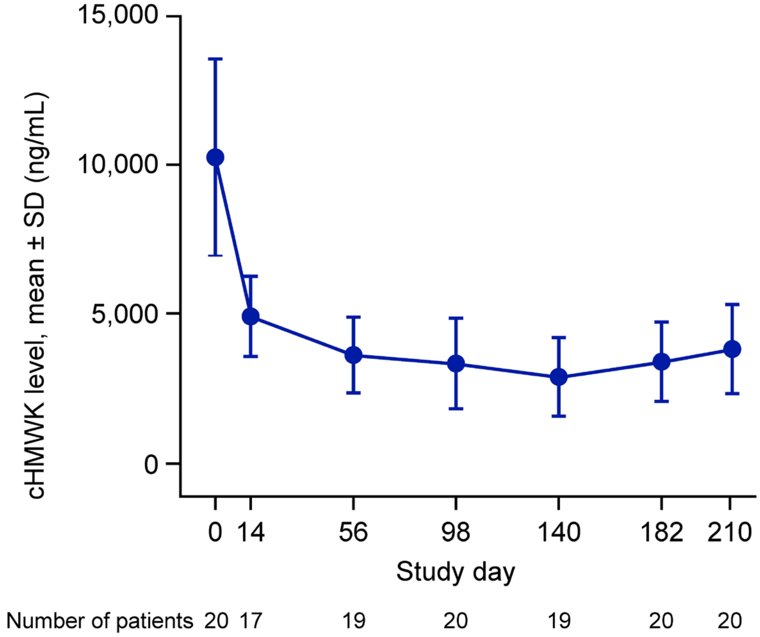

Supplementary Figure 3. Plasma lanadelumab concentration (**A**) and cHMWK levels (**B**) in patients who were ADA positive vs. patients who were ADA negative. ADA, antidrug antibody; cHMWK, cleaved high-molecular-weight kininogen.

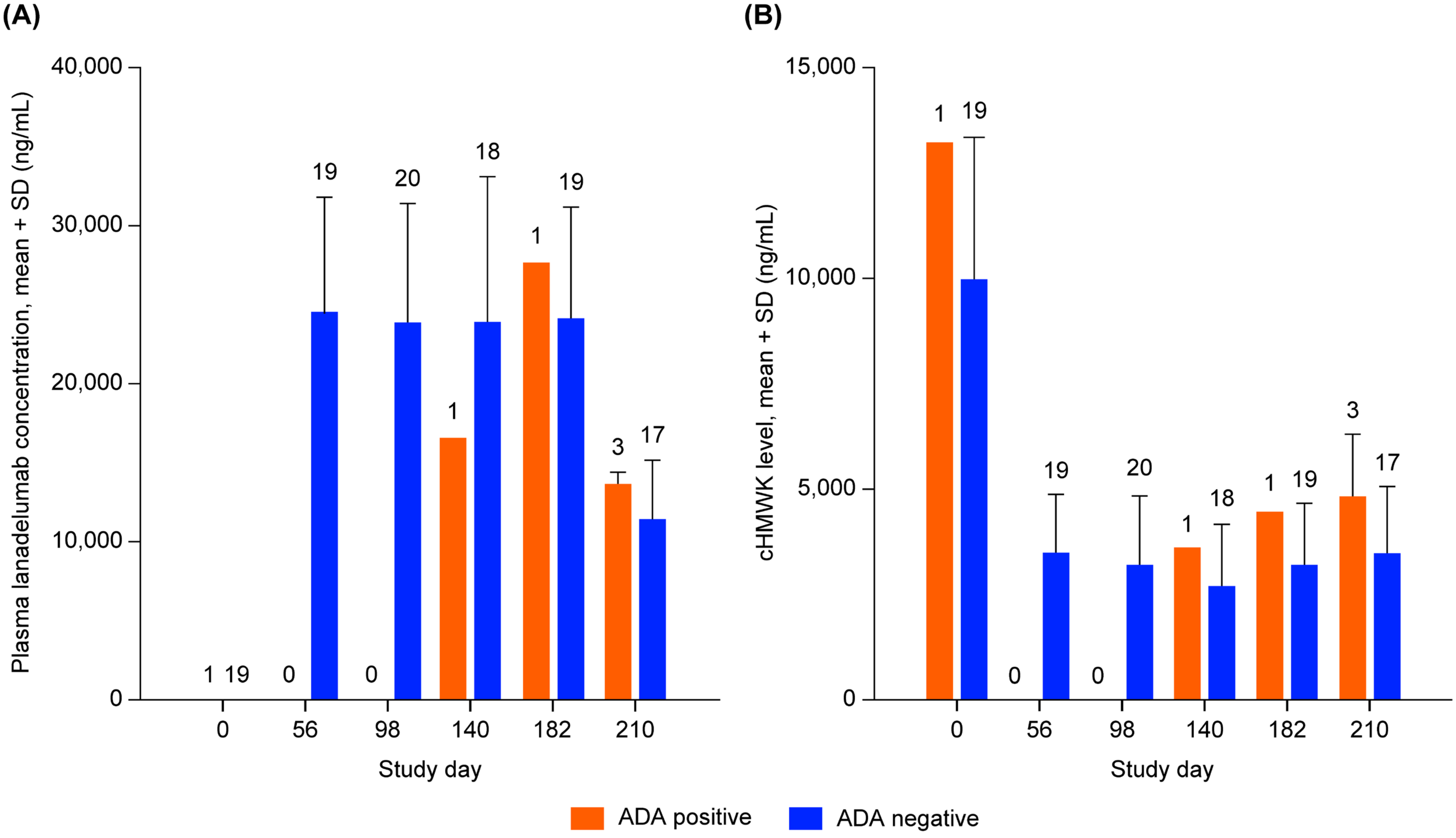

Supplement: Supplementary file 1 [file DataSheet1.pdf]
